# Adverse events associated with nafamostat mesylate and favipiravir treatment in COVID-19 patients

**DOI:** 10.1186/s13054-020-03227-4

**Published:** 2020-08-12

**Authors:** Toru Hifumi, Shutaro Isokawa, Norio Otani, Shinichi Ishimatsu

**Affiliations:** Department of Emergency and Critical Care Medicine, St. Luke’s International Hospital, 9-1 Akashi-cho, Chuo-ku, Tokyo, 104-8560 Japan

**Keywords:** COVID-19, Adverse events, Nafamostat mesylate, Favipiravir

Dear Sir:

I read with interest the article “Nafamostat mesylate treatment in combination with favipiravir for patients critically ill with Covid-19: a case series” by Doi et al. [1]. The authors reported very low mortality rate (9%, 1/11) with the combination nafamostat mesylate + favipiravir irrespective of high rate (73%) of mechanically ventilated patients. Although nafamostat mesylate-related hyperkalemia developed in one patient, adverse events associated with such drug use were not precisely described. As both drugs were developed and used primarily limited to Japan, adverse event cases should be reported from Japan.

We treated 24 laboratory-confirmed coronavirus disease 2019 (COVID-19) patients admitted to the ICU during March 19 to April 30, 2020, in Tokyo. We encountered several complication cases associated with COVID-19 medications, including nafamostat mesylate and favipiravir (not their combination), and recently reported them in emergency medicine journals [2, 3].

Two COVID-19 cases diagnosed with neuroleptic malignant syndrome (NMS) and received favipiravir treatment (not the combination nafamostat mesylate + favipiravir) were recently published. In the letter for our manuscript, Prakash speculated that favipiravir induces serotonin syndrome (SS) probably due to its serotonergic properties [3]. Although the direct association between favipiravir and NMS/SS remains unknown, such central nervous system (CNS) complications require special consideration.

Another complication case was diffuse microbleeding in brain treated with nafamostat mesylate and venovenous extracorporeal membrane oxygenation (VV-ECMO) [2]. A 56-year-old man with severe COVID-19 pneumonia admitted to the ICU received VV-ECMO on day 5 for severe hypoxia. Simultaneously, nafamostat mesylate 200 mg/day was administered for 3 days. Careful coagulation monitoring was performed. He developed cognitive dysfunction; brain computed tomography revealed multiple subcortical hemorrhages. Follow-up cerebral magnetic resonance imaging demonstrated diffuse microbleeding in the subcortical area. Although cerebral hemorrhage causes remain unknown, we should focus on bleeding as nafamostat mesylate is a short-acting anticoagulant.

Therefore, although all three complication cases recovered well, serious adverse events, such as CNS and bleeding complications, require careful consideration when using these drugs in COVID-19 patients. Both efficacy and adverse events should be discussed simultaneously whenever providing new therapy.

## Data Availability

The data of the three patients are available from the corresponding author on reasonable request.

## Abbreviations

COVID-19: Coronavirus disease 2019
NMS: Neuroleptic malignant syndrome
SS: Serotonin syndrome
CNS: Central nervous system
VV-ECMO: Venovenous extracorporeal membrane oxygenation
